# Coffee Consumption and Lung Cancer Risk: A Prospective Cohort Study in Khon Kaen Thailand

**DOI:** 10.31557/APJCP.2020.21.8.2367

**Published:** 2020-08

**Authors:** Wongklang Kudwongsa, Supannee Promthet, Kritika Suwanrungruang, Anakapong Phunmanee, Patravoot Vatanasapt

**Affiliations:** 1 *Doctor of Philosophy Program in Epidemiology and Biostatistics, Faculty of Public Health, Khon Kaen University, Khon Kaen, Thailand. *; 2 *ASEAN Cancer Epidemiology and Prevention Research Group, Khon Kaen, Thailand. *; 3 *Cancer Registry Unit, Faculty of Medicine, Khon Kaen University, Khon Kaen, Thailand. *; 4 *Division of Pulmonology and Critical Care Medicine, Department of Medicine, Faculty of Medicine, Khon Kaen University, Khon Kaen, Thailand. *; 5 *Department of Otorhinolaryngology, Faculty of Medicine, Khon Kaen University, Khon Kaen, Thailand. *

**Keywords:** Coffee, lung cancer, risk factors, cohort

## Abstract

**Background:**

Lung cancer is a major cause of cancer death worldwide. The incidence of lung cancer in Thailand increasing, but risk factors are rarely reported.

**Objective:**

To investigate the effect of coffee consumption on lung cancer in Thai population.

**Methods:**

Between 1990 and 2001, lifestyle and demographic data were collected from 24,528 participants in the Khon Kaen Cohort Study (KKCS), who were followed through 2016, by linking to the Khon Kaen Population-based Cancer Registry. A total of 12,668 eligible participants (68.8% females, mean age 51.0 years at baseline) having complete datasets (239,488 person-years of follow up with 138 incident cases of lung cancer observed) were analyzed using a multi-variable adjusted Cox proportional hazard models.

**Results:**

Coffee consumption was associated with reduced risk for lung cancer (adj. HR = 0.54; 95% CI: 0.35-0.84) after adjusting for age and gender. Cigarette smoking (adj. HR = 2.76; 95% CI: 1.32-5.78) and family history of cancer (adj. HR = 1.65; 95% CI: 1.10-2.48) were associated with higher risk.

**Conclusion:**

This study suggests coffee consumption may be a protective factor for lung cancer in among this cohort.

## Introduction

In 2018, it is estimated that over 2 million new cases of lung cancer were diagnosed globally, while more than 1.7 million deaths occurred the highest among all cancers (Bray et al., 2018). In Thailand, lung cancer is the second leading cause of cancer-related deaths for both men and women, accounting for 4.8% and 3.3% of total deaths, respectively The incidence of lung cancer is highest in the northern region of the country (Wirasorn et al., 2014, 2016; Rankantha et al., 2018; Virani et al., 2017).The average annual percent change (AAPC) from 2000 to 2012 was 3%, but is expected to show decline from 2012 to 2025 (Virani et al., 2017). A well-known risk factor for lung cancer is tobacco smoking (Li et al., 2017). A pooled analysis of Asian cohorts showed tobacco smoking is the strongest risk factor for lung cancer, increasing risk 3- to 4-fold (Zheng et al., 2014). Studies of alcohol drinking, however, have shown limited evidence for smoking-adjusted association with lung cancer risk, with most elevated odds ratios found in hospital-based case-control (Álvarez-Avellón et al., 2017; García Lavandeira et al., 2018). 

Coffee consumption has been studied for its association with lung cancer risk. Generally, coffee consumption has not been shown to be significantly associated with lung cancer risk, but it has been shown to be a risk factor for increased risk among some sub-populations (Xie et al., 2016; Galarraga and Boffetta, 2016; Tang et al., 2010; Narita et al.,2018). Coffee consumption is highly associated with smoking (Guertin et al., 2016). The case-control studies in Thailand reported that chronic exposure to radon and cigarettes smoking increased risk of lung cancer (Pisani et al., 2006; Autsavapromporn et al., 2018). Until presently, no cohort study addressing risk factors of lung cancer in Thai population. Likewise, conflicting evidence was found for the effects of coffee consumption on lung cancer. The effect of coffee has also not been studied. Thus, the aim of this study was to investigate the effects of coffee consumption on lung cancer in Thai population.

## Materials and Methods

*Study design and cancer follow-up*

**Figure 1:**
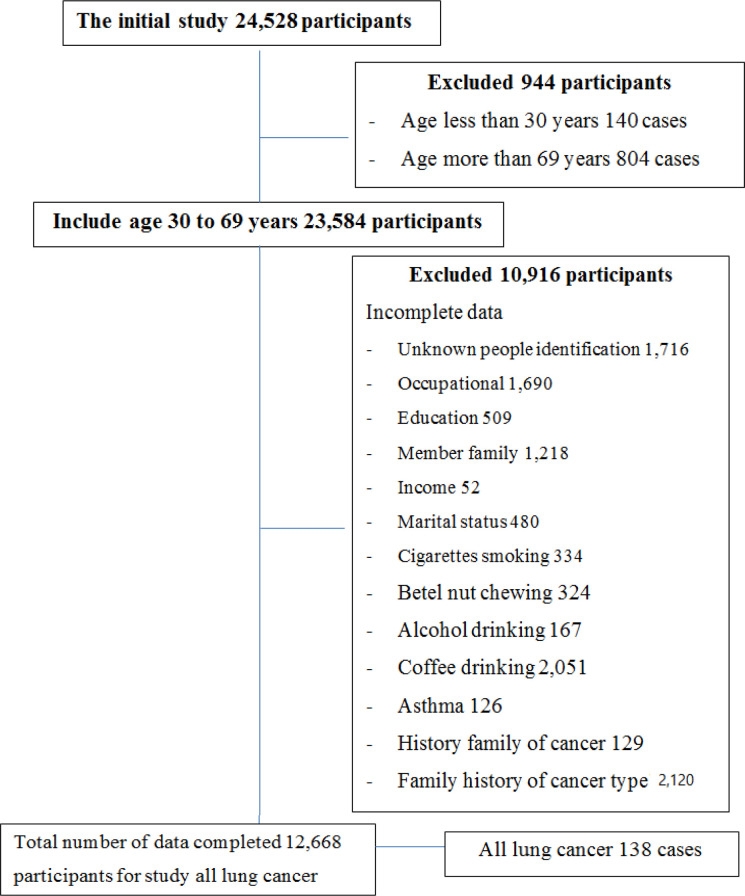
The Flowchart of Study

A prospective cohort was conducted using the data from Khon Kaen Cohort Study (KKCS). The study subjects were residents at Khon Kaen Province enrolled between 1990 and 2001. The inclusion criteria at enrollment were being aged 30-70 years and having no previous diagnosis of cancer. The size of the recruited cohort was 24,528 participants, with 23,584 eligible participants (Figure 1). The exclusion criteria were incomplete data of citizen identification number and the variables to be analyzed. After removing missing data (10,916 participants, 44.5%), the final analysis included 12,668 participants. 

All subjects were followed-up until December 31, 2016 by linking with the population-based cancer registry of Khon Kaen Province to detect lung cancer occurrence using RECLINK-2 program. The vital status of all subjects was retrieved by data linkages with Thai national statistics database. The vital status of all subjects was retrieved by data linkages with the population statistics of the office of the Ministry of Interior using the personal identification number. The completeness of the Thai mortality data is reasonably high except the early infant death (Kijsanayotin et al., 2013). The lung cancer diagnosis was followed the International Classification of Disease for Oncology 3 rd. (ICD-O-3) (C34). 

*Exposure assessment*

All subjects had physical examination at baseline and were interviewed by trained nurses using structured questionnaires. The variables of interests included general information, economic status, coffee consumption, cigarettes smoking, alcohol consumption and history of illness. The definition of coffee consumption is regular drinking of coffee. 

*Statistical analysis*

To evaluate the relation between coffee consumption, other factors, and the risk of lung cancer, crude hazard ratios (CHR), adjusted hazard ratios (Adj.HR) with their 95 percent confidence intervals (95%CI), and p-values were estimated using a multi-variable adjusted Cox proportional hazard models. Person-years in the cohort were calculated from baseline until cancer diagnosis, death, or loss to follow-up. 

Prior to analysis, continuous variables were categorized into two groups Tests of homogeneity of subjects were done using Pearson’s Chi-squared test. Bivariate crude analyses were used to identify factors associated with lung cancer for inclusion in the model (p-value ≤ 0.25 computed by Wald’s test). Backward stepwise elimination method was used to remove non-significant factors (p<0.05). P-values were tested two-sided with values <0.05 considered significant. The assumption test of proportional hazard model was test for goodness of fit and test for fit model. All analyses were conducted in STATA program version 15.0 (copyright Faculty of Public Health, Khon Kaen University).

*Ethical considerations*

This research was approved by The Ethical Committee for Human Research of Khon Kaen University, Thailand (HE611216).

## Results

Of 12,668 subjects, mean age 50.96 years, most of them were female (68.8%), married (85.2%), employed in agriculture, had a primary school education, lived in a nuclear household, and had a household income less than 10,000 THB/month (around 1,000 USD in 2020)(Table 1). The total follow-up was 239,488 person-years. Lung cancer was diagnosed in 138 cases (57.63 cases per 100,000 per year). The most common histology types were adenocarcinoma (33.33%), followed by squamous cell carcinoma (7.25 %), large cell carcinoma (5.07%), and small cell carcinoma (0.72%), with the remaining cancers classified as unspecified malignant neoplasm (49.28%). 

*Factors associated with lung cancer*

We found the risk of lung cancer was higher in males, cigarette smokers, those older at enrollment, and those with a family history of cancer (Table 2). Cigarette smoking demonstrated trends whereby increased exposure (e.g., less filtration or higher frequency was associated with increased risk of lung cancer (Table 2). No association was found for BMI or alcohol drinking. Coffee consumption was found to be a protective factor for Lung cancer (adj. HR = 0.54; 95% CI: 0.35-0.84). 

The final multivariable model confirmed that coffee consumption was associated with reduced risk of lung cancer (Adj.HR=0.54; 95% CI: 0.35-0.84). It also confirmed the risk of lung cancer from cigarette smoking (adj. HR = 2.76; 95% CI: 1.32-5.78) and family history of cancer was likely to increase risk of lung cancer (adj. HR = 1.65; 95% CI: 1.10-2.48) (Table 3).

**Table 1:** The General Characteristics of the Study Subjects in the Cohort

Variables	N= 12,668	%
Sex		
Male	3,950	31.18
Female	8,718	68.82
Age at recruitment		
30-39 Years old	1,101	8.69
40-49 Years old	4,517	35.66
50-59 Years old	4,874	38.47
60-70 Years old	2,176	17.18
Mean (S.D.)	50.96 (±8.08)	
Marital status		
Single	260	2.05
Married	10,808	85.32
Divorced or widowed	1,600	12.63
Occupation		
Agriculture	10,928	86.27
Business owner	497	3.92
Factory worker	12	0.09
Laborer	136	1.07
Government officer	15	0.12
General employee	116	0.92
Unemployed	964	7.61
BMI (kg/m^2^) (n=10,070)		
< 18.5	626	6.22
18.5 - 22.9	3,800	37.74
23 - 24.9	1,966	19.52
≥25	3,678	36.52
Family size		
Nuclear Family	8,976	70.86
Extended family	3,541	27.95
Big family	151	1.19
Family income/month (Baht)		
<10,000	12,216	96.43
10,001-20,000	319	2.52
20,001-30,000	90	0.71
>30,000	43	0.34

**Table 2:** The Factors Associated with Lung Cancer

Factors	Number (N=12,668)	Lung cancer	Crude HR (95%CI)	*P* value
Sex				<0.001
Female	8,718	54	1	
Male	3,950	84	3.51 (2.49-4.94)	
Age at recruitment				0.002
30-49 Years old	5,618	46	1	
50-70 Years old	7,050	92	1.74 (1.22-2.48)	
BMI (kg/m^2^)				0.011
Normal weight	3,800	60	1	
Low weight	626	9	0.91 (0.45-1.84)	
Overweight/Obese	5,644	49	0.57 (0.39-0.83)	
Missing data	2598			
History of family cancer				0.027
No	10,148	97	1	
Yes	2,520	41	1.54(1.06-2.22)	
Coffee Consumption				0.161
No	8,896	106	1	
Yes	3,772	32	0.76 (0.51-1.13)	
Cigarette Smoking				<0.001
No	9,386	61	1	
Yes	3,282	77	3.72 (2.66-5.20)	
Alcohol drinking				0.001
No	6,719	56	1	
Yes	5,949	82	1.75 (1.25-2.46)	
Betel nut chewing				0.738
No	10,771	119	1	
Yes	1,897	19	0.92 (0.57-1.49)	

HR, Hazard ratio; 95% CI, 95 percent confident interval; *P*-value from partial likelihood ratio

**Table 3:** Final Multivariate Model of Significant Factors Independently Associated with Lung Cancer

Factors	Numbers	Lung cancer	Crude HR (95%CI)	Adjusted HR* (95%CI)	*P*-value**
Coffee Consumption					0.006
No	8,896	106	1	1	
Yes	3,772	32	0.76 (0.51-1.13)	0.54 (0.35-0.84)	
Cigarette Smoking					0.007
No	9,386	61	1	1	
Yes	3,282	77	3.72(2.66-5.20)	2.76(1.32-5.78)	
History of family cancer					0.014
No	10,148	97	1	1	
Yes	2,520	41	1.54(1.06-2.22)	1.65(1.10-2.48)	

HR bases on backward elimination by cox proportion hazard regression; *HR, hazard ratio; adjusted for sex, age, and BMI; **p-value from Wald test.

## Discussion

This study is the first cohort study on risk factors of lung cancer in Thailand. We found an inverse association between coffee consumption and lung cancer, with risk reduction of at least 16%. Reduced risk of lung cancer has been identified among smokers who drink black tea (Mendilaharsu et al., 1998), and among smokers drinking decaffeinated coffee (Baker et al., 2005). Coffee consumption has also been associated with decreased risk of other cancers, such as bladder, breast, and colorectal cancers (Yu et al., 2011).

Coffee contains some biochemical compounds, polyphenols as the main bioactive, which has been found to be an antioxidant (Wierzejska, 2017), as well as catechins and flavonoids that found to be have chemoprotective effects such as promoting inhibiting oxidative stress, regulation of DNA repair, apoptosis, anti-inflammation, antiproliferative, oxidative damage, antiangiogenetic effects and antimetastatic effects (Baker et al., 2005; Bøhn et al., 2014). High intake of flavonoids, another compound found in coffee, also can reduce risk of lung cancer. Flavonoids intake about 20 mg/day decreased risk of lung cancer by10% (Tang et al., 2009; Palmioli et al., 2017) Chlorogenic acid (CGA), an important biochemical active in of polyphenol found in coffee, affects inhibition of COX-2, MMP-2, and MMP-9 expression, which eventually reduced the risk of tumor enlargement and metastasis (Bułdak et al., 2018). CGA may also have anti-oxidative effects (Tajik et al., 2017).

This study used a large community-based prospective cohort and data linkage to the population-based cancer registry to ensure the temporality on its association. However, this study also has several limitations. First, only half of cancer cases were diagnosed with histopathological confirmation. But remaining cases were diagnosed with imaging which were verified for diagnosis of lung cancer in the standard procedure for cancer registry. Second, the information on exposure relied on onetime interview at their enrollment without follow up data. Third, the data on type of coffee consumption (black coffee or mixed), and the amount of glass per day were not data collected at the beginning of projected. The future study on this topic should have more data collection detail. The recorded behaviors might have changed afterward causing non-differential misclassification. This would weaken association between those factors and lung cancer, but it would less likely to mislead their effects. The study also excluded a large proportion of eligible participants, which may have caused selection bias. As a result, the final analysis included demographics (e.g., 68.8% female) that may not necessarily represent the community.

In conclusion, this study suggests that coffee consumption may be a protective factor for lung cancer among this cohort. Future study of coffee consumption with histological types of lung cancer in a larger sample sizes is needed. Further study on the effects of the biochemical compounds within coffee should also try to identify possibly mechanisms to explain protective effects on lung cancer.
